# Comparison of Vitamin D and Resveratrol Performances in COVID-19

**DOI:** 10.3390/nu15112639

**Published:** 2023-06-05

**Authors:** Cristina Russo, Maria Stella Valle, Luisa Malaguarnera, Ivana Roberta Romano, Lucia Malaguarnera

**Affiliations:** 1 Section of Pathology, Department of Biomedical and Biotechnological Sciences, School of Medicine, University of Catania, 95123 Catania, Italy; cristina.russo@unict.it (C.R.); luisamalaguarnera92@gmail.com (L.M.); lucmal@unict.it (L.M.); 2 Section of Physiology, Department of Biomedical and Biotechnological Sciences, School of Medicine, University of Catania, 95123 Catania, Italy; ivanarobertaromano@yahoo.it

**Keywords:** COVID-19, vitamin D, resveratrol, inflammation, anti-oxidant activity, anti-thrombotic activity

## Abstract

Over the last few years, we have experienced the infection generated by severe respiratory syndrome coronavirus 2 (SARS-CoV-2) often resulting in an exaggerated immune reaction and systemic inflammation. The preferred treatments against SARS-CoV-2 were those that mitigated immunological/inflammatory dysfunction. A variety of observational epidemiological studies have reported that vitamin D deficiency is often a crucial factor in many inflammatory diseases and autoimmune diseases, as well as the susceptibility to contract infectious diseases, including acute respiratory infections. Similarly, resveratrol regulates immunity, modifying the gene expression and the release of proinflammatory cytokines in the immune cells. Therefore, it plays an immunomodulatory role that can be beneficial in the prevention and development of non-communicable diseases associated with inflammation. Since both vitamin D and resveratrol also act as immunomodulators in inflammatory pathologies, many studies have paid particular attention to an integrated treatment of either vitamin D or resveratrol in the immune reaction against SARS-CoV-2 infections. This article offers a critical evaluation of published clinical trials that have examined the use of vitamin D or resveratrol as adjuncts in COVID-19 management. Furthermore, we aimed to compare the anti-inflammatory and antioxidant properties linked to the modulation of the immune system, along with antiviral properties of both vitamin D and resveratrol.

## 1. Introduction

Severe respiratory syndrome coronavirus 2 (SARS-CoV-2) also known as coronavirus disease 2019 (COVID-19) spread rapidly, causing chaos for about three years reaching, and almost 7 million deaths worldwide. Because of its emerging variants, rapid dispersion, severity, and treatment options, COVID-19 remains a global health problem. SARS-CoV-2 infection is highly contagious because of the droplets that are spread by talking, coughing, or sneezing [1]. The disease symptoms are mild or moderate in most affected individuals. Infection with SARS-CoV-2 significantly affects the respiratory system. Some patients develop a severe form of the illness, ranging from severe acute respiratory syndrome (SARS), acute lung injury (ALI), pulmonary fibrosis, and multisystem organ failure due to dysregulated coagulation cascade, and, ultimately, septic shock [1]. Beyond causing viral pneumonia, COVID-19 has an impact on the cardiovascular system. Both patients with potential cardiovascular risk factors (diabetes mellitus, obesity, dysmetabolic diseases, old age) and patients with proven cardiovascular disease constitute a vulnerable population in terms of increased morbidity and mortality during COVID-19. Cardiovascular problems resulting from COVID-19 infection involve acute coronary artery syndrome, pulmonary thromboembolism, myocarditis, and possible arrhythmias. Extra-pulmonary symptoms include gastrointestinal and renal manifestations [2], and serious psychological manifestations such as depression, stress disorders, and mild cognitive impairment [3]. Moreover, among the associated complications of COVID-19, a variety of neurological manifestations have been widely described in the central and peripheral nervous systems [4]. The most common manifestations concern headaches, dizziness, sleep disturbances, and taste and smell impairments. Neuropathological variations have also been observed in the brains of COVID patients. Notably, an MRI study found cerebellum volume loss in these patients [5]. Normally, the cerebellum is fundamental to the maintenance of a functional gait [6]; therefore, cerebellar damage can impair motor adaptation and learning. This would explain why damage to the motor system can include unsteady gait, muscle ache/injury, fatigue, and also abnormal deep reflexes, which are frequently encountered. Under normal conditions, deep reflexes can adapt to individual situations [7]; instead, patients with COVID-19 may have a great variety of reflex behaviors, with accentuated hyperreflexia [8], or conversely, areflexia syndrome [9]. In several subjects, an additional manifestation is the “long COVID” sequelae [3,10]. The early phase in SARS-CoV-2 infection is the link between the COVID-19 virus and the host cell receptors, consisting of angiotensin-converting enzyme 2 (ACE2) and the dipeptidyl peptidase-4 (DPP4) receptor. Among ACE2-related enzymes promoting the entry of SARS-CoV-2 include peptidases, are alanyl aminopeptidase (ANPEP) and glutamyl aminopeptidase (ENPEP) [11]. Additionally, transmembrane serine protease 2 (TMPRSS2) also enables the entrance of SARS-CoV-2 into host cells. An exaggerated inflammatory response, deregulated host immune systems, and abnormal levels of pro-inflammatory cytokines such as tumor necrosis factor-alpha (TNF-α), interleukin-6 (IL-6), interleukin-1 (IL-1), interferon-gamma (IFN-γ), monocyte chemoattractant protein-1 (MCP-1), granulocyte colony-stimulating factor (G-CSF), and granulocyte–macrophage colony-stimulating factor (GM-CSF), are responsible for pathogenicity and the severity of COVID-19 [12]. Thus, treatment designed to specifically resolve a ‘cytokine storm’ and inflammation response by the use of immunomodulators can be among the main goals in improving the course of this infection. Adjuvant therapy to fortify the immune system in the course of infection has been regarded as an effective, affordable, and easily accessible alternative. To improve the outcome of COVID-19 infection, many alternative therapies have been tried, even the experimental use of stem cells/allogeneic mesenchymal stromal cells (MSC) administered intravenously and secretome derived from MSCs with immunomodulatory/anti-inflammatory/regenerative properties [13,14,15]. Moreover, the literature and experimental evidence has showed that both vitamin D and resveratrol play an immunomodulatory role and they have been explored for their therapeutic potential in the avoidance or management of COVID-19 [16]. Some clinical trials have reported that supplementation of vitamin D was effective in preventing infections in the initial and in the hyper-inflammatory phases of the illness, since the supplementation positively modulated the immune response versus SARS-CoV-2. In humans, vitamin D, a sterol derivative, is principally obtained from exposure to ultraviolet rays (UVR) or from food sources (fish or mushrooms) [17]. A zoosterol, 7-dehydrocholesterol, is photochemically transformed in the skin into pre-vitamin D following exposure to UVR. The first step of vitamin D conversion occurs in the liver. In this organ, cholecalciferol is hydroxylated to 25-hydroxy-VitD (25(OH)D), by the cytochrome P450 hydroxylase enzymes, CYP27A1 and CYP2R1. Afterwards, in the kidneys, a further hydroxylation by the cytochrome P450 enzyme (CYP27B1) transforms 25(OH)D into the active 1,25-dihydroxyvitamin D3 or calcitriol. Moreover, vitamin D is also triggered in several cellular types by CYP27B1, as well as in the immune system cells, where it regulates a plethora of cellular functions. Calcitriol interacts with its vitamin D receptor (VDR) and, subsequently, with vitamin D response elements (VDREs), modulating gene transcription. Currently, there are conflicting opinions regarding the impact of vitamin D supplementation in relation to the severity and mortality of COVID-19 illness. Resveratrol belongs to the polyphenolic family, more specifically, to the stilbenoids [18]. It is a compound contained in *Vitis vinifera* (Vitaceae), *Morus nigra* (Moraceae), in several fruits, and in peanuts [19]. Resveratrol is the most studied stilbenoid because of its wide range of pharmacological activities [19]. Additionally, this compound influences the immune response by acting as an antiviral, antioxidant and anti-inflammatory agent [19,20]. To date, it has been proven that resveratrol inhibits SARS-CoV-2 spreading within mammalian cell cultures, and it has been proposed as a phototherapeutic treatment for this viral infection [21]. In this review, we aimed to compare the anti-inflammatory and antioxidant properties linked to the modulation of the immune system, along with the cytotoxicity and antiviral properties of vitamin D and resveratrol, to highlight their importance as a co-adjuvant treatment applied to integrative complementary medicine in this pandemic viral infection.

## 2. ACE2 Receptor and SARS-CoV-2 Infection

As previously mentioned, the spike protein of SARS-CoV-2 infects the target cells via the binding to the membrane protein, ACE2, and co-receptor, TMPRSS2. ACE2 is largely found in the epithelial cells of the trachea, bronchi, alveoli, in alveolar monocytes and macrophages, in mucosal cells of the intestines and tubular epithelial cells of the kidneys, in cerebral neurons, immune cells, endothelial cells of veins and arteries [17,22]. Many of the clinical symptoms such as respiratory failure and liver, gastrointestinal, and renal damage are caused to the presence of ACE2 in these cells. Above all, macrophages, which are recruited from the SARS-CoV-2-infected target cells by the CD74/MIF interaction, can have both protective and damaging functions. Most lung cells expressing ACE2 are alveolar epithelial cells of type II (AECII), therefore, since these cells serve as a repository for viral invasion, the lung is the most susceptible organ [23]. AECII expressing ACE2 facilitates coronavirus replication as a consequence of an increase in genes that regulate the viral life cycle [24].

The spike protein bound to the membrane protein, ACE2, represents the essential condition for transmission and begins the association between COVID-19 and the renin-angiotensin–aldosterone system (RAAS). The RAAS is an endocrine system implicated in short-term blood pressure regulation through the increase in total peripheral resistance mediated by angiotensin II (Ang II), and, in the long term, through the reduction of renal excretory function that makes to move to the right within the renal pressure natriuresis curve. Like all systems that govern homeostasis, the RAAS can be involved in a regulation–counter-regulation model in which the trigger of a specific cascade produces a response of self-regulation and suppression. The effects of angiotensin II are mediated by binding to two specific receptors: angiotensin II type 1 receptor (AT1R) and angiotensin II type 2 receptor (AT2R). Most of its actions, including vasoconstriction, stimulation of aldosterone release, and increased renal tubular sodium reabsorption, as well as many deleterious effects such as fibrosis and inflammation, are facilitated by AT1R. Conversely, the activation of signal transduction pathways that underlie the AT2R receptor and the Mas receptor, generally counteract the classical actions of AT1R-mediated action, producing vasodilation and natriuresis as a protective counter-regulation system. In animal models of acute respiratory distress syndrome (ARDS), activation of the Ang II-AT1R axis is linked to the severity of lung damage, whereas both increased ACE2/AT (1–7) and initiation of the Mas receptor pathway, neutralize these deleterious effects [25]. The pro-renin receptor inhibition decreases the formation of interstitial edema, hemorrhagic phenomena, the number of neutrophils, and the level of activated pro-renin in rat lungs [26]. In addition, ACE2 overexpression reduces ARDS induced by LPS through the Ang-(1–7)/MasR pathway, avoiding activation of the extracellular, signal-regulated kinase/nuclear factor kappa B (NF-κB) [27]. Therefore, since ACE2 would counteract acute lung injury, its inhibition due to SARS-CoV-2 might cause ARDS. Deepening the pathophysiological mechanisms triggering the evolution of lung and systemic damage during infection with SARS-CoV-2 is an essential requirement for the therapeutic management of the disease and the development of effective treatment, both with direct antiviral and immunomodulatory effects.

## 3. Vitamin D and SARS-CoV-2 Infection

**Figure 1:**
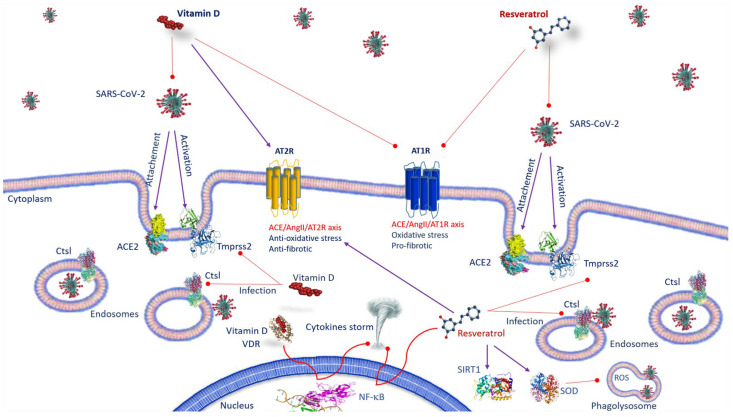
Anti-SARS-CoV-2 mechanisms of action of vitamin D and resveratrol. Vitamin D, by inhibiting the binding of ACE2 to the spike protein and by decreasing TMPRSS2 and Ctsl gene expression, prevents the transmission of SARS-CoV-2. As well as vitamin D, resveratrol affects the expression of ACE-2, suppresses the effects AT1R axis, improves AT2R axis and prevents SARS-CoV-2 entry, inhibiting Ctsl and TMPRSS2. Both vitamin D/VDR complex and resveratrol inhibit NF-κB activation and cytokine storm. Resveratrol, activating SIRT1 and SOD, decreases inflammation. Abbreviations: ACE2—angiotensin-converting enzyme 2; AT1R—angiotensin II type 1 receptor; AT2R—angiotensin II type 2 receptor; Ctsl—Cathepsin L; NF-κB—nuclear factor kappa B; ROS—reactive oxygen species; SARS-CoV-2—severe respiratory syndrome coronavirus 2; SIRT1—sirtuin 1; SOD—superoxide dismutase; TMPRSS2—transmembrane serine protease 2; VDR: vitamin D receptor.

Vitamin D deficiency (VDD) is usually observed in patients developing ARDS. VDD is likely to promote pulmonary fibrosis by activating the RAAS [28]. In considering vitamin D performance, it is important to remember the role of calcium. Low calcium intake is often associated with VDD [29]. In coronavirus infections, calcium is important to mediate the fusion of the viral envelope with the host cell membrane or from the S1/S2 viral subunit or from the ACE2 receptor transmembrane domain [2]. This generates the altered conversion of angiotensin II (Ang-II) to angiotensin 1–7 and the origin of a cytokine storm as well as tissue injury. Vitamin D is able to regulate the function of endothelial cells and, consequently, vasodilation. These processes facilitate ARDS onset [2]. Thus, vitamin D safeguards from lung injury by preventing the expression of renin and the axis of the ACE/angiotensin II type I receptor (AT1R) plus stimulating the ACE2/Ang receptor (1–7)/Mas G (G-protein-Mas receptor pair) [30]. It has been shown that, in addition, vitamin D decreases the gene expression of TMPRSS2 and Cathepsin L (Ctsl) in treated mice [31]. Ctsl is an endosomal cysteine protease that facilitates S protein cleavage and supports viral infection through virus–host cell endosome membrane fusion [32]. Ctsl inhibitors prevent the access of pseudotyped SARS-CoV-2 in humanized mice [33]. Moreover, genome-wide CRISPR screening has shown that Ctsl is crucial for SARS-CoV-2 entrance [34]. This result suggests that vitamin D impedes SARS-CoV-2 access and, thus, can moderate or prevent SARS-CoV-2 infection in lung epithelial cells [31]. The vitamin D/VDR signaling pathway acts favorably in LPS-mediated respiratory distress syndrome by reducing the cytokine storm and by regulating the RAAS in COVID-19 patients [35]. Moreover, it has been found that vitamin D and VDR, by the negative regulation of the RAAS cascade, and inhibition of NF-κB and Wnt/β-catenin [36], decrease the permeability of the epithelial–endothelial barrier and counteract fibrosis in the lungs, liver, and kidneys [36]. This action prevents the lung injury induced by sepsis, obstructing the pathway of the Angiopoietin-2-TEK receptor, tyrosine-myosin light chain kinase. Likewise, certain vitamin D metabolites are able to bind various enzymes of the SARS-CoV-2 virus replication machinery involved in the maintenance of the infection, preventing their action. Paricalcitol, a vitamin D analogue, alleviates ALI induced by LPS, preventing NF-κB activation as well as Ras expression, a member of the homologous A/Rho kinase family signaling pathways [2]. Furthermore, vitamin D and its active hydroxyderivatives can avoid the binding of ACE2 to the receptor binding domain of SARS-CoV-2, thus avoiding SARS-CoV-2 infection. Hence, a treatment involving the integration of these compounds could be an encouraging therapeutic strategy for COVID-19 (Figure 1) [37].

## 4. ACE2 Receptor and Resveratrol and SARS-CoV-2 Infection

Resveratrol affects the regulation of the RAAS and the expression of ACE-2. It suppresses the detrimental effects of the angiotensin II (Ang II)/angiotensin II type 1 (AT1R) receptor axis and improves the AT2R/angiotensin 1–7 (Ang 1–7)/Mas receptor axis, defending against renal aging [38]. The effect of resveratrol on the two opposite pathways in the renin–angiotensin system (RAS) may equally be useful in COVID-19 patients [20]. It has recently been found that resveratrol oligomers could prevent SARS-CoV-2 access by inhibiting Ctsl activity [16]. Nevertheless, since Wang et al. [16] observed that the active cavity of Ctsl is quite large for resveratrol, they suggested that resveratrol might avoid SARS-CoV-2 entry also inhibiting TMPRSS2. Then, it is fair to assume that Ctsl could be a valuable target for treating COVID-19. Resveratrol exerts its beneficial effects mainly through the activation of sirtuin 1 (SIRT1) [39]. Stimulating both SIRT1 and superoxide dismutase (SOD), it promotes an increase in ACE2 function and a decrease in inflammation biomarkers [40,41]. Among the many involvements of SIRT1 in cellular processes are inflammation, stress response, and apoptosis regulation [42]. Therefore, the upregulation of ACE2 by resveratrol may be useful in preventing SARS-CoV-2 infection and avoiding the progression of severe forms of the disease. In adipose tissue where ACE2 is significantly expressed, supplementation with resveratrol decreases both ACE2 and leptin levels, a pro-inflammatory adipokine [43]. These effects following resveratrol supplementation could make obese individuals less susceptible to SARS-CoV-2 and could exert a beneficial impact on the outcome of the disease [43]. On the basis of this evidence, it is worth examining the dosage range in which resveratrol has the most evident influence on the expression of ACE2, as well as the effectiveness of resveratrol supplementation on viral load and spread, and on the occurrence of COVID-19 in populations at risk, such as in obese people or those with metabolic diseases [43]. Thus, it is important to investigate the mechanisms of action of resveratrol on the gene expression of ACE2 and the clinical outcomes of COVID-19 during infection (Figure 1).

## 5. The Immune Response against SARS-CoV-2 Infection

**Figure 2:**
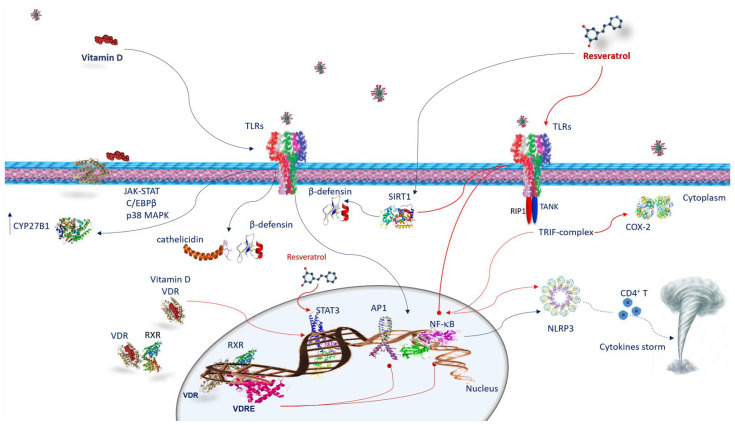
Vitamin D and resveratrol immune response against SARS-CoV-2 infection. Vitamin D interacts with the cells of the innate immunity by activating TLRs and upregulating CYP27B1. The mechanism by which TLR binding improves CYP27B1 expression involves JAK-STAT, C/EBPβ, and p38 MAPK pathways. Following TLRs stimulation, vitamin D activates innate immunity, increasing cathelicidins and β-defensins. VDR is expressed in many immune cells. Once vitamin D is hydroxylated interacts with VDR, vitamin D/VDR complex negatively regulates STAT3. Vitamin D exerts its effects through genomic mechanisms modulated by VDR/RXR complex to bind to VDRE in target genes of immune cells such as neutrophils, macrophages, dendritic cells, and T and B lymphocytes. VDRE promotes the recruitment of nuclear proteins in transcriptional-complex-modulating inflammatory response. Resveratrol acts against COVID-19 inhibiting TRIF signaling in the TLRs pathway by RIP1/TANK in the TRIF complex. It inhibits STAT3 phosphorylation. Resveratrol reduces COX-2 expression by preventing TRIF signaling. Resveratrol also activates the SIRT1 pathway, which disrupts TLR4/NF-κB/STAT signal, decreasing cytokine storm. Resveratrol-inducing SIRT1 can mediate β-defensins induction. Both vitamin D and resveratrol inhibit NF-κB and consequently, NLRP3 inflammasome, avoiding CD4^+^ T cell activation and cytokine storm. Abbreviations: AP1—activator protein 1; C/EBPβ—CCAAT/enhancer binding protein β; CD4T—CYP27B1, cytochrome P450 family 27 subfamily B member 1; COX2—cyclooxygenase-2; JAK-STAT—Janus kinase/signal transducer and activator of transcription; NF-κB—nuclear factor kappa B; NLRP3—NLR family pyrin-domain-containing 3; p38 MAPK—p38 mitogen-activated protein kinase; RIP1—receptor-interacting protein; RXR—retinoid X receptor; SIRT1—sirtuin 1; STAT 3—signal transducer and activator of transcription 3; TANK—TRAF family member-associated NFKB activator; TLR—Toll-like receptor; TRIF—TIR-domain-containing, adapter-inducing interferon-β; VDR—vitamin D receptor; VDRE—vitamin D response elements.

Innate immune cells recognize the pathogen-associated molecular patterns (PAMPs) and damage-associated molecular patterns (DAMPs) of virus particles through pattern recognition receptors (PRRs) such as Toll-like receptors (TLRs). Thus, identifying viral particles activates the innate immune system and triggers local inflammation liberating cytokines, IL-1β, IL-2, IL-17, G-CSF, GM-CSF, TNF-α, and chemokines such as MCP1, and IP-10, IL-8, and MIP1α (or CCL3). The cytokine storm supports a great infiltration of neutrophils and macrophages, causing severe alveolar injury [2]. Additionally, T cells, CD4^+^, Th1 and Th17, and CD8^+^ T cells are the key players in the adaptive immune response, particularly in fostering the production of pathogen-specific antibodies by inducing T-dependent B cells and in the elimination of virus-infected cells. SARS-CoV-2-specific CD4^+^ T cells produce IL-2, IFN-γ, and tumor necrosis factor-α (TNF-α), increasing the activation of Th1 immune response and cell-mediated immunity (Figure 2) [2], whereas virus-specific memory CD8^+^ T cells are involved in host defense from SARS-CoV-2 infection by the secretion of IFN-γ, TNF-α, IL-2, and cytolytic molecules such as granzyme B [44].

## 6. Anti-Inflammatory Activity and Immune Response of Vitamin D and Resveratrol against SARS-CoV-2 Infection

Both vitamin D and resveratrol, constraining the secretion of pro-inflammatory cytokines, exert anti-inflammatory activity, and play an immunomodulatory role in both adaptive and innate immune systems [2,17,45] (Figure 2). Notably, cells of the immune system such as neutrophils, monocytes, macrophages, dendritic cells, B cells CD4^+^, and CD8^+^ T cells express VDR; hence, they are targets of vitamin D. Additionally, most of them, through VDR, are able to activate and inactivate vitamin D metabolites [45]. Likewise, resveratrol activates macrophages, T cells, and natural killer (NK) cells, and is involved in the CD4^+^CD25^+^ regulatory T cell immunosuppressive role [19].

### 6.1. Vitamin D and Innate Immune Response against SARS-CoV-2 Infection

The actions of vitamin D on the immune system are largely determined by the vitamin D receptor (VDR). VDR belongs to the family of nuclear steroid receptors and is expressed in tissues of the whole body, including many immune cells [2], and affects many cellular functions. VDR operates by binding with its ligand [1α,25(OH)2D3] and creating a heterodimer with the retinoid X receptor (RXR). The VDR/RXR complex, by binding the proximal-promoter VDR response element (VDRE), promotes the enrollment of nuclear proteins in the transcriptional complex and modulates gene expression. The interplay of vitamin D with the cells of the innate immune system occurs through the activation of TLRs. It has been found that VDR-binding 1,25(OH)_2_D_3_ can promote TLR2/1 heterodimer ligation in macrophages, upregulating CYP27B1 [2]. The CYP27B1 gene provides instructions to generate an enzyme called 1α-hydroxylase. Human 1α-hydroxylase promoter analysis has identified binding sites for various transcription factors such as NF-κB, AP1, AP2 and Sp1; cAMP binding sites (CRE); a CCAAT box; and a GAS.3 site [46]. TLR binding enhances CYP27B1 expression, involving JAK-STAT, C/EBPβ, and p38 MAPK pathways [47]. The activities of these factors can be controlled by inflammatory stimuli. Several TLRs, such as TLR2, TLR3, TLR4, TLR6, TLR7, TLR8, and TLR9 are potential targets in controlling the early stages of COVID-19 infection [48]. It has been shown that vitamin D induces TLR2 and TLR4 [2,45]. Moreover, vitamin D regulates TLR9-dependent IL-6 production and increases TLR7 and NE/PAD4/COX-3/GAPDH [49]. In human macrophages, as a result of TLR2/1 stimulation, vitamin D activates innate immunity increasing the antimicrobial peptide levels of cathelicidins and β-defensins (Figure 2) [17]. These findings strongly suggest that individuals with sufficient vitamin D levels are able to counteract TLRs and subsequent AMPs and have adequate cytokine production [17]. 

### 6.2. Vitamin D and Adaptive Immune Response against SARS-CoV-2 Infection

Vitamin D powerfully influences macrophage polarization. In particular, it blocks the polarization from the M1 phenotype towards the M2 by decreasing IFN-γ release and promoting IL10 production [2]. The M2 phenotype inhibits inflammation, stimulating type 2/Th2 immune responses and producing IL-10, which in turn, prevents the M1 polarization. [2]. This is also fundamental in the conversion of pro-inflammatory Th1 cells versus regulatory T cells (Tregs) to resolve inflammation in severe COVID-19 through the production of TGF-β, CCL22 and additional IL-10 [2]. Therefore, the production of vitamin D by macrophages results in a modification from an inflammatory to a tolerogenic state [2]. Interestingly, vitamin D, increasing IL-4, IL-5, and IL-10 and reducing Th17 cells and IL-17 production, upregulates the Th2-specific transcription factors, GATA-3 and c-maf. In addition, IL-17 reduction inhibits the inflammatory response dominated by neutrophils. Th17 cell proliferation is induced by signals mediated by IL-6 IL-21, IL-23, TGF-β, and by the retinoic-acid-related orphan nuclear receptor (RORγT), which is a lineage-specifying transcription factor. Other crucial targets of vitamin D are dendritic cells (DCs) that belong to antigen-presenting cells (APCs), which affect lymphocyte activation, prompting the adaptive immune response and maintaining peripheral tolerance (Figure 2) [2]. Remarkably, the vitamin D–VDR complex binds to the promoter of STAT3, IL-6, and IL-10 genes. Furthermore, vitamin D, influencing T cell responses, indirectly acts on B cells. Since B cells express VDR and CYP27B1, these cells are also predisposed to vitamin D effects, including the inhibition of their proliferation and plasma cell differentiation, and the induction of apoptosis. Consequently, vitamin D is capable of modulating antibody production [2]. 

### 6.3. Resveratrol and Innate Immune Response against SARS-CoV-2 Infection

Recent evidence has demonstrated that resveratrol strongly prevents viral replication of both DNA and RNA viruses [50,51]. An in vitro investigation also demonstrated that resveratrol decreases viral replication of SARS-CoV-2 and prevents cytotoxicity [50]. A randomized, double-blind clinical trial confirmed that resveratrol can efficiently serve as a treatment adjunct for COVID-19 [52]. In silico examinations reported that resveratrol is able to bind to the ACE2 complex [53]. Resveratrol is also able to decrease the effect of proinflammatory cytokines such as IFN-γ, TNF-α, and IL-1β, which are important factors in the onset of the cytokine storm in COVID-19 [20]. The anti-inflammatory action of resveratrol has been linked to the suppression of NF-κB activation [54] by facilitating the inhibition of Iκ-B kinase [55]. The NF-κB pathway is strongly associated with the pathogenesis of numerous inflammatory diseases and the manifestation of severe inflammatory sequelae [56]. It is well known that an exaggerated activation of NF-κB elicits the evolution of the inflammatory response with the production of many proinflammatory mediators such as IL-1, IL,6, TNF-α, IFN-γ, interleukin-8 (IL-8), granulocyte-macrophage-colony-stimulating factor (GM-CSF), COX-2, and inducible nitric oxide synthase (iNOS) [57]. Numerous immunosuppressive or anti-inflammatory agents usually target the NF-κB pathway [58]. In addition, NF-κB stimulates the activation of the NLR family pyrin domain containing the 3 (NLRP3) inflammasome by facilitating the priming signal of the inflammasome. The NLRP3 inflammasome induces the maturation and secretion of pro-inflammatory cytokines, ultimately promoting CD4^+^ T cell differentiation and activation [59]. Therefore, the inhibitory effect of resveratrol on NF-κB results in the decline of the expression of inflammation-associated genes, supplying a suitable target for anti-inflammatory treatments (Figure 2) [17]. It has been found that resveratrol reduces COX-2 expression by preventing TIR-domain-containing, adapter-inducing interferon-β (TRIF) signaling associated with TLR3 and the TLR4 MyD88-independent pathway through TRAF family member-associated NF-κB activator (TANK)-binding kinase 1 and receptor-interacting protein 1 (RIP1) in the TRIF complex [17]. Thus, it is possible that the action of resveratrol against SARS-CoV-2 occurs through specific inhibition of TRIF signaling in the TLR3 and TLR4 pathways by involving kinase 1 and the RIP1 TANK binder in the TRIF complex. However, resveratrol activates other anti-inflammatory pathways, including Sirt1 [17]. This latter inhibits the TLR4/NF-κB/STAT signal, which, in turn, diminishes cytokine release from inactivated immune cells [17], or macrophage/mast-cell-derived pro-inflammatory factors, as the platelet-activating factor, histamine and TNF-α [60]. Other targets of resveratrol are pathways influencing immune function and the cellular inflammation response. For example, it can cause the conversion of cellular metabolism and signal transduction pathways by distressing enzymes, such as adenosine monophosphate kinase and the serine/threonine protein kinase that is the mechanistic target of rapamycin (mTOR) [58]. Overall, the antiviral mechanisms of resveratrol versus SARS-CoV-2 can act through the inhibition of virus replication, synthesis of proteins, and inhibition of transcription and signaling pathways, along with viral-related gene expressions [17]. Some evidence has indicated that resveratrol, in response to microbial agents such as *Haemophilus influenzae* and *Streptococcus pneumoniae* [61,62], is capable of inducing the production of several classes of antimicrobial peptides (AMPs) such as α- and β-defensins and cathelicidin [63]. Therefore, it is conceivable that resveratrol and its analogues, through SIRT1 protein, also mediate β-defensin induction following SARS-CoV-2 infection. Resveratrol, reestablishing glutathione concentrations, can interfere with the differentiation of monocytes to macrophages and inflammation (Figure 2) [64]. Moreover, if on the one hand, resveratrol regulates the M2 macrophage activation and differentiation impeding IL-10 and MCP-1 productions, on the other, it promotes TGF β1 production [17]. Nevertheless, resveratrol inhibits the phosphorylation of STAT3 without interfering with its expression in the M2 macrophage differentiation. 

## 7. Vitamin D and Resveratrol Effects on Neutrophil Extracellular Traps (NETS) in SARS-CoV-2 Infection

In severe COVID-19 patients, the number of neutrophils increases [65]. Neutrophils are phagocytic cells that, with their microbicide activity, their degranulation mechanisms, and their release of neutrophil extracellular traps (NETs), play an essential role in the defense against infectious diseases. NETs are web-like chromatin structures consisting of cytosolic and granule proteins that are collected on a support of uncondensed chromatin. NETs originate from the nucleus but also contain mitochondrial DNA [66]. NETs contain abundant enzymatic proteins such as neutrophil elastase, myeloperoxidase, and peptidyl arginine deiminase 4. Therefore, NETs have antibacterial activity able to eradicate any kind of microbial agent [66]. Some COVID-19 patients have exhibited elevated levels of NETs [67]. Therefore, their dysregulation is implicated in the COVID-19 pathogenesis. NETs promote cytokine release, initiating a signal cascade of the inflammatory type that causes micro-thromboses and pulmonary, cardiovascular, and renal system complications [68]. It has been observed that vitamin D can control the proliferation of pathogens, such as viruses, by inducing NETs [69]. However, the role played in the initiation of NETs by vitamin D in SARS-CoV-2 appears to be ambivalent [2,70]. In contrast, it has been found that resveratrol reduces NET arrangement in severe COVID-19 patients mitigating neutrophil activation [71].

## 8. Oxidative Stress and Thrombosis in COVID-19 Patients

Severe COVID-19 infection is usually related to coagulopathy and thrombosis as a result of endothelial injury [72]. The pro-inflammatory status, the establishment of a cytokine storm, and the imbalance between an excessive formation of reactive oxygen species (ROS) and antioxidative mechanisms are some of the principal causes of vascular endothelial impairment. A damaged vascular endothelium, in turn, is crucial to trigger hypoxemia thrombosis [73]. Hyper-coagulopathy and thrombosis are significant protagonists in COVID-19 [74], and are distinctive traits of comorbidities, such as obesity, diabetes, atherosclerosis, and hypertension, frequently linked with serious implications in COVID-19 [75].

## 9. Anti-Oxidant and Anti-Thrombotic Activity of Vitamin D in COVID-19 Patients

Physiopathological inflammation and hemostasis are two interrelated processes. They act mutually in a positive feedback loop involving several factors, including chemokines, adhesion molecules, pro-inflammatory cytokines, tissue factor expression (TF), and pro-coagulant mediators [76]. Overall, these mediators trigger endothelial dysfunction, platelet reactivity, coagulation cascade initiation, suppression of natural anticoagulant pathway defeat, and fibrinolytic activity [76]. A fundamental consequence of the inflammation response is the generation of oxidative stress. Oxidative stress arises if ROS production oversteps the antioxidant capacity of the cell. Hence, it is indispensable to determine the vitamin D and resveratrol influences in the pathophysiology of thrombosis. Enhanced intracellular oxidative stress is able to activate the NLRP3 inflammasome [77]. Several scientific investigations have demonstrated that the vitamin D/VDR signaling pathway constrains tissue damage and reduces the activation of inflammation and the role of the NLRP3 inflammasome [57]. VDR represses caspase activation, production of ripe IL-1β, and BRCC3-mediated pyroptosis in human tubular epithelial cells [78]. VDR engaging NLRP3 avoids the inflammasome function in human macrophages [78]. One of the main transcription factors increasing the antioxidant potency of cells is the nuclear factor, erythroid-2-related factor 2 (Nrf2) [79]. The promoter region of the Nrf2 gene comprises a portion of a gene, a “response element”, that binds VDR [80]. It has been found that the vitamin D–VDR complex restrains ROS-mediated activation of the NLRP3 inflammasome across Nrf2 nuclear translocation, which induces its transcriptional activity for enzymes inhibiting oxidative stress and DNA damage [80]. Amplified oxidative stress can affect platelet function. Platelet dysfunction and their consequent adhesion to the endothelium of the vascular system—generated by a wide range of mediators including proinflammatory cytokines, chemokines, adhesion molecules, complements, and procoagulant factors—results, in turn, in endothelial dysfunction, increased leukocyte and platelet reactivity, and activation of coagulation. Vitamin D plasma levels are inversely related to the mean platelet volume, a marker for platelet activity [81]. Activated platelets induce the CD40 protein (CD40-L) to bind with its CD40 receptors present on the platelets, causing their activation. Moreover, CD40-L binding to CD40 on vascular endothelial cells induces a great expression of specific proteins, the intercellular and vascular adhesion molecules (ICAM and VCAM), and the production of chemokine CCL2, which determines the migration and activation of leukocytes and further ROS production [82].

**Figure 3:**
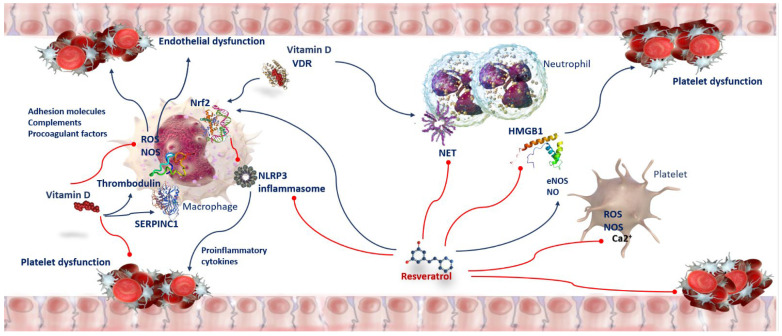
Anti-oxidant and anti-thrombotic activity of Vitamin D and Resveratrol in COVID-19. Oxidative stress affects platelet function. Adhesion molecules, complements, and procoagulant factors trigger endothelial dysfunction, increased leukocyte and platelet reactivity, and activation of coagulation. Vitamin D/VDR prevents ROS-mediated activation of NLRP3 by Nrf2 translocation to the nucleus, inducing its transcriptional activity for antioxidant enzymes to inhibit oxidative stress. Vitamin D increases the levels of SERPINC1 transcripts and antithrombin. Resveratrol moderates ROS and NOS, inhibits arachidonic acid metabolism and hampers Ca^2+^ entry into platelets. Resveratrol exerts antithrombotic properties by increasing the activity of nitric oxide endothelial synthase (eNOS) and NO levels. Resveratrol-induced NO production results from its direct interaction with SIRT1 and Nrf2. Resveratrol exerts anti-inflammatory effects inhibiting HMGB1-mediated signaling pathway. Abbreviations: Ca^2+^—Calcium ion; eNOS—nitric oxide endothelial synthase; HMGB1—high-mobility group box 1; NET—neutrophil extracellular traps; NLRP3—NLR family pyrin-domain-containing 3; NOS—nitric oxide synthase; Nrf2—nuclear factor erythroid-2-related factor 2; ROS—reactive oxygen species; SERPINC1—serpin family C member 1; VDR—vitamin D receptor.

Numerous studies point to an inverse relationship between vitamin D status and thrombotic episodes [83,84]. Indeed, the antithrombin gene has several vitamin-D-response elements. In silico analysis has revealed that the serpin family C member 1 (*SERPINC1*) gene contains potential vitamin-D-response elements (VDRE) [85]. The vitamin D pathway is important for the regulation of *SERPINC1,* and this data is supported by the HepG2 cell line. In fact, Paricalcitol, a vitamin D analogue, increases the levels of antithrombin and *SERPINC1* transcripts liberated into the secretome of this cell line [85]. It has been suggested that genetic variations in SERPINC1 VDRE could exacerbate the consequences of vitamin D deficiency (Figure 3) [85]. 

Observations establishing an antithrombotic role of vitamin D/VDR signaling have been confirmed by transcriptomic analysis, in which it has been shown that in human monocytes, the vitamin D/VDR signaling pathway involves the thrombomodulin gene [86]. However, there are controversies in the argument. Investigations report that vitamin D supplementation reduces thrombotic phenomena, others claim that it increases them [87,88]. In addition, vitamin D supplementation in non-deficient individuals does not condition their plasma thrombogenic profile, in some cases, vitamin D supplementation can increase the likelihood of bleeding events [89]. Therefore, it is indispensable to detect the vitamin D supplementation effect in carriers of polymorphisms of these genes. Further investigations are required to estimate the potential benefits of vitamin D in COVID-19 immunothrombosis (Figure 3). 

## 10. Anti-Oxidant and Anti-Thrombotic Activity of Resveratrol in COVID-19 Patients

Resveratrol has powerful antioxidant properties (Figure 3). It reduces biomarker levels of oxidative stress and affects hemostasis. In human blood platelets, oxidation triggered by peroxynitrite (ONOO^−^) is formed in the reaction between superoxide anion (O_2_^−^) and nitric oxide (NO). Resveratrol pre-treatments protect against the oxidation of thiols such as glutathione, cysteine and cysteinylglycine [90]. It has been found that it protects from alteration of oxidative and nitrative protein and from lipid peroxidation [91]. It also moderates ROS levels in platelets activated by thrombin [92]. Moreover, by inhibiting NADPH oxidase-derived ROS generation and consequent oxidative deactivation of SH2 domain-containing protein tyrosine phosphatase-2, it exerts an anti-platelet action. Resveratrol-suppressing platelet aggregation affects primary hemostasis [93]. Resveratrol reduces nitric oxide concentration, inhibits arachidonic acid metabolism in platelets in vitro and cyclooxygenase activity [94], decreases cytoplasmic Ca^2+^, and hampers Ca^2+^ entry into blood platelets. Modulating the clotting through the suppression of tissue factor/factor VIIa complex formation can prevent secondary hemostasis [95]. Likewise, Pterostilbene (a dimethyl ether analogue of resveratrol) and isorhapontigenin (a novel derivative of stilbene) show anti-platelet properties. The first occurs through preventing platelet aggregation in response to collagen and decreasing P-selectin exposition [96]. The second occurs by inhibiting blood platelet activation stimulation by ADP, through the P2Y12 receptor [97]. Resveratrol, with its important anti-thrombotic properties, is able to slow the complications of severe COVID-19, such as vascular thrombosis [98]. In addition to the abovementioned mechanisms, it has been suggested that the antithrombotic properties of resveratrol also arise from its ability to promote the increase in NO levels by increasing the activity of nitric oxide endothelial synthase (eNOS), responsible for NO formation in vascular endothelium [99]. Modulation of NO levels avoids and constrains the serious lung complications of COVID-19 [100]. NO, besides being a valid anti-inflammatory and vasodilator, also has antiviral properties and, therefore, is capable of inhibiting SARS-CoV-2 replication [101]. Resveratrol-induced NO production results from its interface with intracellular pathway components including SIRT1, protein-kinase-activated adenosine monophosphate, and Nrf2 [99]. Ultimately, resveratrol acts by promoting Nrf2 activation by reducing Keap-1 expression reduction and stimulating SIRT1 deacetylase [102]. After the activation of Nrf2, together with Keap1, it split off from the complex and initiates some nuclear target genes transcription such as antioxidant response element (ARE), avoiding cell inflammation, oxidative stress, and thrombotic events [103]. Resveratrol also exerts anti-inflammatory effects through the suppression of the high-mobility group box 1 HMGB1-mediated signaling pathway [104]. HMGB1 is another important inductor of thrombosis via platelet activation, promotion of inflammatory processes, neutrophil recruitment, generation of micro-thrombosis, and NET development (Figure 3) [105]. HMGB1-induced thrombosis is linked to ARDS evolution in severe SARS-CoV-2 infections [106].

## 11. Concluding Remarks

**Figure 4:**
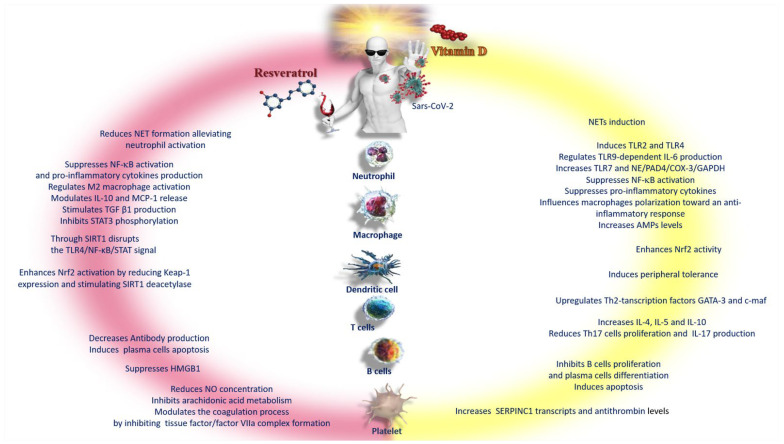
Schematic representation of the differences between vitamin D and resveratrol in the response to the inflammatory phenomena of COVID-19. Abbreviations: AMPs—adenosine monophosphate; c-maf—c-transcription factor Maf; COX-3—cyclooxygenase-3; GAPDH—glyceraldehyde-3-phosphate dehydrogenase; GATA-3—GATA binding protein 3; HMGB1—high-mobility group box 1; IL—interleukin; MCP—monocyte chemoattractant protein; NE—neutrophil; NET—neutrophil extracellular traps; NF-κB—nuclear factor kappa B; Nrf2—nuclear factor erythroid-2-related factor 2; PAD4—peptidyl arginine deiminase-4; SERPINC1—serpin family C member 1; SIRT1—sirtuin 1; STAT 3—signal transducer and activator of transcription 3; TGFβ1—transforming growth factor beta 1; Th-2—lymphocytes; TLR—Toll-like receptor.

In this review, analyzing the similarities and differences of the molecular mechanisms used by vitamin D and resveratrol in the battle against SARS-CoV-2 infection, it has become apparent that both are not only powerful immunomodulatory agents but also behave as enzymatic inhibitors, since they use the same mechanisms of interaction against the virus (Figure 4). We found only a few slight differences between the signal pathways used. In some cases, the use of resveratrol has been found to be less ambivalent than vitamin D. For example, in reducing the formation of NETs or as an anti-thrombotic, it could be a more effective adjuvant [69,71]. From the differences, an obvious question arises: is it possible to use both to make up for the small shortcomings in the mechanisms manifested by both, individually, in order to achieve a synergistic effect?

Unfortunately, patients in several countries still cannot access expensive antiviral drugs and are frequently treated only symptomatically, and occasionally with nutritional adjuvants in the form of vitamins, minerals, and polyphenolic compounds. These compounds have been proven to be valuable adjuvants in supporting antiviral treatment and in promoting faster recovery times and improved survival. Therefore, alternative strategies using synergistic combinations, in order to increase their specificity and activity at low cost, are desirable. 

The beneficial impact of vitamin D and resveratrol is not confined only to regulating the immune response against COVID-19. Precisely because they are effective anti-inflammatory adjuvants, both could be of great value in attenuating a large number of manifestations related to long COVID-19, such as cardiovascular, neurological, and motor system disorders [107,108,109]. This is particularly applicable to at-risk subgroups, such as obese individuals with dysmetabolic diseases and elderly individuals.

Although the pandemic has just been declared over, it is necessary to investigate the action of these compounds in management support not only for long COVID-19 but also for similar future diseases.

## Data Availability

The data that support this review derive from articles published in PubMed from 1999 to 2023.
